# Retraction Note: Second-order convergence analysis for Hall effect and electromagnetic force on ternary nanofluid flowing via rotating disk

**DOI:** 10.1038/s41598-024-61759-z

**Published:** 2024-05-14

**Authors:** Faisal Shahzad, Wasim Jamshed, Sayed M. El Din, Md. Shamshuddin, Rabha W. Ibrahim, Zehba Raizah, Adnan

**Affiliations:** 1 Department of Mathematics, Capital University of Science and Technology (CUST), Islamabad, 44000 Pakistan; 2 Center of Research, Faculty of Engineering, Future University in Egypt, New Cairo, 11835 Egypt; 3 Department of Mathematics, School of Sciences, SR University, Warangal, 506371 Telangana India; 4 Mathematics Research Center, Department of Mathematics, Near East University, Near East Boulevard, PC: 99138 Nicosia, Mersin 10 Turkey; 5 Department of Mathematics, College of SCIENCE, King Khalid University, P. O. BOX 62529, Abha, Saudi Arabia; 6 Department of Mathematics, Mohi-Ud-Din Islamic University, Nerian Sharif, 12080 AJ&K Pakistan

Retraction of: *Scientific Reports* 10.1038/s41598-022-23561-7, published online 05 November 2022

The Editors have retracted this Article.

After publication concerns were raised about the use of nonsensical phrases, the citation of references that are not relevant and contribution to authorship. The authors have not provided satisfactory responses to these concerns. The Editors therefore no longer have confidence in the results and conclusions presented.

Md. Shamshuddin and Rabha W. Ibrahim have not explicitly confirmed whether they agree or disagree with this retraction. Faisal Shahzad, Wasim Jamshed, Sayed M. El Din, Zehba Raizah and Adnan have not responded to correspondence from the Editors about this retraction.

